# Knowledge, attitude and control practices of sickle cell diseases among senior secondary students in Osun State, Nigeria

**DOI:** 10.11604/pamj.2021.38.350.20894

**Published:** 2021-04-12

**Authors:** Adeyemo Deborah Oluwadamilola, Temidayo Ifeoluwa Akinreni, Moshood Adekunle Adefisan, Seun Deborah Olayiwola

**Affiliations:** 1 Department of Community Medicine, Osun State University, Osogbo (Main Campus), Osun State, Nigeria,; 2 Ondo State Primary Health Care Development Agency, Akure, Ondo State, Nigeria

**Keywords:** Sickle cell disease, knowledge, attitude, secondary schools

## Abstract

**Introduction:**

sickle cell disease is one of the greatest public health problems of this age. This study was conducted to assess the knowledge, attitude and control practices on sickle cell disease (SCD) among selected secondary school students in Osun State, Nigeria.

**Methods:**

a descriptive cross-sectional study involved 420 secondary school students within Osogbo Metropolis selected by a multistage stratified sampling technique, using self-administered structured questionnaire. Data were collected using pre-tested self-administered semi structured questionnaire. Data were analyzed using SPSS version 20.

**Results:**

a total of 420 students were interviewed, modal age range 15-20 years. There were more females (55%) than males (45%). Majority of them were christians (57.1%). A larger percentage of the respondents were aware of SCD (58.5%). However, comprehensive knowledge as regards the various genotypes related to SCD, tests to be done for genotype screening among the respondents is low. One third of the respondents had positive attitude towards SCD (65%) and nearly one half (48%) of the respondents had bad control practices.

**Conclusion:**

findings in this study shows a high level of general awareness on SCD, even though comprehensive knowledge as regards the various genotype related to SCD, tests to be done for genotype screening among others is low. The need to improve on their attitude and practice towards the disease is highly recommended because having a good knowledge is not as important as applying the knowledge in a way to stop the spread of the disease.

## Introduction

Sickle cell disease (SCD) affect the blood cells, causing incidents of 'sickling' which produce incidents of pain and other signs. In between incidents of sickling, people with sickle cell disease are normally well. Long-term crises can occur [1]. Acute painful sickle cell incidents (also known as painful crises) are triggered by obstruction of the small blood vessels. The red blood cells in sickle cell patient behave differently under a variety of conditions including dehydration, low oxygen levels and high temperature or cold. Changes in any of these conditions may cause the cells to block small blood vessels and cause tissue infarction. Frequently occurrence of the incidents may result in organ damage [2]. The first description of sickle cell disease was given by a Chicago physician, James B Herrick, who noted in 1910 that a patient of his from the West Indies had an anemia characterized by unusual red cells that were sickle shaped [3]. In 1927, Hahn and Gillespie showed that sickling of the red cells was related to low oxygen. In 1940, Sherman (a student at Johns Hopkins Medical School) noted a birefringence of deoxygenated red cells, suggesting that low oxygen altered the structure of the hemoglobin in the molecule [4]. This made sickle cell disease the first disorder in which an abnormality in a protein was implicated [5].

The following are the types of sickle cell disease; Hemoglobin SS disease, Hemoglobin SC disease, Hemoglobin SB+ (beta) thalassemia, Hemoglobin SB 0 (Beta-Zero) thalassemia, Hemoglobin SD, Hemoglobin SE, Hemoglobin SO. Of the above types of sickle cell diseases Hemoglobin SS is the most common type [6,7]. In the previous study done, it was discovered that there is a low knowledge as regard management and prevention of SCD despite a high level of awareness of the disease among the study population [8], also another study by [9], shows that 81.8% of the respondents claimed to have heard about sickle cell disease (SCD) but only 38.0% of them knew the cause of SCD. The study by [10] discovered that majority of the student in the senior secondary school are in premarital phase and do not know their hemoglobin genotype, they also have a poor understanding of the disease and some will stigmatize patients with sickle cell disease.

## Methods

This is a descriptive cross-sectional study was conducted among students in public and private secondary schools within Osun State, Nigeria. A single population proportion formula was used to determine the sample size using Leslie Fischer´s formula. Prevalence estimates of sample size was calculated based on the findings from a similar study which showed a prevalence of 50% [8]. Using 95% confidence level and 5% margin of error, the required sample size was 384 participating students. Participants were selected using a multi-stage sampling technique. At the first stage, from the list of secondary schools in Osogbo, Osun State, two local governments were chosen by simple random sampling. Then the list of all registered secondary schools (both public and private) in the selected local government areas (LGAs) was obtained from the ministry of education. Three private and three public secondary schools in each of the selected LGAs were selected by simple random sampling via balloting technique, making a total of 6 schools. The desired sample size was selected using stratified random sampling with proportional allocation of respondents from the different classes in the selected secondary schools; stratification was along the line of classes (senior secondary (SS) 1 to senior secondary (SS) 3).

Ethical approval was obtained from the Health Research Ethics Committees (HREC), Osun State University, Osogbo. Permission to conduct the study was also obtained through the permanent secretary of Osun State Ministry of Education. Additionally, participants´ consent was sought verbally before proceeding with the data collection. Quantitative data was collected from the participants using a pretested semi-structured self-administered questionnaire that was purposely designed to seek information about the students´ knowledge, attitude and control practices of sickle cell disease. The data was analyzed using SPSS 20.0 and appropriate univariate, bivariate and multivariate analyses were done at a significance level of less than or equal to 0.05.

## Results

There are 420 respondents who completely filled the questionnaire, of which 130 (31%) are at the range of age 10-14 years, 288 respondents (68.8%) are between the age of 15-19 years, while 2 (0.5%) are of age 20-24. There are 189 (45%) male and 231 (55%) are female. Data was collected from senior secondary school students of which 252 (60%) are from SS1 and 168 (40%) from SS2. Two hundred and forty (57.1%) are christians while 180 (42.9%) were muslims. Four hundred and seventeen (99.3%) are single while 3 (0.7%) are married. Four hundred (95.2%) are Yoruba while 10 (2.4%) are Igbo 2 (0.5%) are Hausa while 8 (1.9%) are other ethnicity. In terms of fathers´ occupation, 181 (43.1%) are professionals, 80 (19%) are skilled workers while 159 (37.9%) are unskilled workers. Regarding mothers occupation 111 (26.4%) are professional while 45 (10.7%) are skilled workers while 264 respondents mother (62.9%) are unskilled workers. Two hundred and thirteen (50%) fathers are degree holder, while 150 father (35.7%) fathers are senior secondary certificate of education (SSCE) holder, 10 (2.4%) fathers are primary school holder, 29 (6.9%) fathers have adult literacy education while 18 (4.3%) fathers have no formal education, also 180 (42.9%) mothers are degree holder, 168 (40%) mothers are SSCE holder, 17 (4%) mothers are primary school holders, 41 (9.8%) mothers have adult literacy education and 14 (3.3%) mothers have no formal education (Table 1).

**Table 1:** socio-demographic characteristics of respondents

Variables		Frequency	Percentage (%)
Age groups (years)	10-14	130	31.0
	15-19	288	68.6
	20-24	2	0.5
Gender	Male	189	45.0
	Female	231	55.0
Class level	Ss1	252	60.0
	Ss2	168	40.0
Religion	Christianity	240	57.1
	Islam	180	42.9
Marital status	Single	417	99.3
	Married	3	0.7
Ethnicity	Yoruba	400	95.2
	Igbo	10	2.4
	Hausa	2	0.5
	Others	8	1.9
Fathers occupation	Professional	181	43.1
	Skilled labor	80	19.0
	Unskilled labor	159	37.9
Mothers occupation	Professional	111	26.4
	Skilled	45	10.7
	Unskilled	264	62.9
Fathers highest level of education	No formal education	18	4.3
	Adult literacy	29	6.9
	Primary	10	2.4
	Secondary	150	35.7
	Tertiary	213	50.7
Mothers highest level of education	No formal education	14	3.3
	Adult literacy	41	9.8
	Primary	17	4.0
	Secondary	168	40.0
	Tertiary	180	42.9

Of all the 420 respondents, 246 (58.6) were aware of SCD while 174 (41.4) were not aware of SCD. In terms of definition, 183 (43.6%) gave correct answers regarding SCD definition while 62 (14.8%) gave incorrect answers while 175 (41.7%) have no idea of SCD definition. Majority of the respondents´ source of information about SCD include school 153 (36.4%), television (TV) 41 (9.8%), internet, social media platform 28 (6.7%) and radio 24 (5.7%). Concerning questions regarding whether SCD can be transmitted, 255 (60.7%) agreed that SCD can be transmitted while 165 (39.3%) said SCD can´t be transmitted. Finally, they were asked of the mode of transmission and 6 (14%) got it correctly while 248 (59%) were incorrect. Concerning whether SCD can be detected in the body, majority of them agreed that it can be detected in the body 367 (87.3%) while 53 (12.6%) disagreed (Table 2). Concerning respondents´ attitude towards SCD in this study. Out of the 420 respondents, 272 (65%) have a positive attitude towards SCD while 148 (35%) have negative attitude towards SCD.

**Table 2:** knowledge on sickle cell disease

Variables		Frequency	Percentage (%)
Aware of SCD	Yes	246	58.6
	No	174	41.4
If yes, what is SCD (n=246)	Correct answer	183	43.6
	Incorrect answer/no ideal	62	14.8
What is your source of information	Health education	153	36.4
	Radio	24	5.7
	Television	41	9.8
	Internet, social platform	28	6.7
Can SCD be transmitted	Yes	255	60.7
	No	165	39.3
If yes, mode of transmission (n=255)	Correct answer	6	1.4
	Incorrect answer/no ideal	248	59.0
Can SCD be detected in somebody	Yes	367	87.4
	No	53	12.6

In terms of control practices, over two-fifth 230 (54.7%) of the respondents had done genotype screening while 190 (45.3%) had not done genotype screening before. Similarly, as regards the participants´ genotype, 177 (42.1%) were AA, 41 (9.8%) AS, 9 (2.1%) AC, 3 (0.7%) SS. In response to where they had their screening test, 207 (49.3%) had their screening in the hospital while 21 (5%) did theirs in the laboratory, 31 (7.4%) in schools. Majority of the schools do not have SCD club 389 (92.6%), and the few who have engage their students in activities such as awareness creation (1.4%), genotype testing (1.5%), counseling (1%), sensitization (0.7%) and seminar (0.7%). Furthermore, few of them 33 (7.8) are not interested in participating in activities to stop the spread of SCD. Reasons given by respondents´ who do not have interest in any activities to stop the spread of SCD were inadequate time 28 (6.7%), parent approval 3 (0.7%) not interested 28 (6.7%) and discrimination 1 (0.2%) (Table 3).

**Table 3:** control practices related to sickle cell disease among respondents

Variables		Frequency	Percentage (%)
Have you done genotype screening before	Yes	230	52.9
	No	197	54.2
If yes, what is your genotype (n=230)	AA	177	42.1
	AS	41	9.8
	AC	9	2.1
	SS	3	.7
Where was the test done (n=230)	Hospital	209	49.3
	Diagnostic center/lab	21	5.0
Do you have sickle cell club in your school	Yes	31	7.4
	No	389	92.6
If yes, what are the activities of the club (n=31)	Awareness creation	6	1.4
	Genotype testing	8	1.9
	Counseling	4	1.0
	Sensitization	7	0.7
	Seminar /outreach	6	0.7
I will you like to participate in any activity to stop the spread of the SCD	Yes	387	92.1
	No	33	7.8
If no, why not (n=33)	Time	28	6.7
	Parents approval	3	0.7
	Not interested	1	6.7
	Discrimination	1	0.2
If yes, which of the activities will you want to be involved in the campaign to reduce occurrence of SCD (n=387)	Awareness creation	95	22.6
	Genotype testing	157	37.4
	Counseling	72	17.1
	Sensitization	21	5.0
	Seminar/outreach	40	9.5
	Others	2	0.5

## Discussion

Sickle cell disease (SCD) is the most prevalent genetic disease [1,8,11]. This study assesses the knowledge, attitude and control practices of sickle cell disease among senior secondary school student in Osogbo Osun State. The respondents´ ages ranged from 10-24 years with the modal age group of 15 + 4 years. There were more females (55%) than males (45%). Majority of them were Christians (57.1%). Over two-third of the respondents fathers and mothers had at least secondary school education. This is similar to the study done on the knowledge and attitudes of secondary school students in Federal Capital Territory (FCT), Abuja, Nigeria [9] on sickle cell disease in 2011 where 80.7% fathers and 70.2% mothers of the respondents had at least secondary school education, in regards to family history of SCD. Also two-third of the respondents claimed not to have any history of SCD.

Furthermore, knowledge on SCD is high as over half of them claimed to be aware of SCD but less than one-third of them could define the disease correctly. The high level of awareness may be due to the fact that SCD and other related education is being taught in social studies in the junior secondary school thereby making school an important source of information, this finding is similar to the result gotten by [8,12] in their study on major source of information include schools (36.4%), radio (5.7%), TV (9.8%), and internet/social media platform (6.7%). This shows that the lessons taught in social studies on SCD and related topics in secondary schools is of good effect on students´ knowledge about SCD in relation with other studies [9,11,13].

Moreover, complementing the high level of awareness, but comprehensive knowledge on important topics such as diagnosis, dangers and benefits of genotype screening, when to do genotype screening is low among respondents this is different to the result gotten by previous researchers on SCD [9,12,14] and this could be due to difference in time and area of study as awareness on premarital genotype screening has increased compared to the period of previous studies. About three quarter gave correct answers to when genotype screening should be done, as well as dangers and benefits of doing the test. In summary, more than two third of respondents had good knowledge on SCD while one quarter had poor knowledge. Due to a high level of awareness and knowledge among the respondents, it was discovered during the course of carrying out our study that over half of the respondents have a favorable attitude towards SCD as over three quarter of respondents disagreed to the abortion of unborn SCD babies and a similar percentage also agreed to making genotype screening mandatory in secondary schools. About three quarter disagreed to SCD screening being a waste of time and over half of the respondents disagreed to discouraging premarital genotype screening in churches and mosques, a similar fraction also disagreed to the opinion that SCD screening would expose their genetic status to the public.

About half of the respondents had done genotype before, 42.1% are AA, 9.8% are AS, 2.1 are AC and 0.7% are SS. This is different from results gotten by [8] as (81%) of their respondents had done genotype testing before and 59.2% were AA, (11.1) AS, (29.6%) had other combinations, the difference in this result is believed to be due to difference in area of study. Furthermore, less than one quarter of the respondents have sickle cell club in their school and over three quarter of them are willing to participate in activities to stop the spread of SCD. Over half of the respondents have good practices towards SCD. Comparing social demographic factors and summarized knowledge score, it was discovered that gender as well as religion and parents´ occupation plays a significant role in the knowledge of respondents on SCD. The male respondents have a good knowledge on SCD compared to the females, and the Christians have good knowledge than the Muslims. Also, respondents whose parents are professional workers have good knowledge about SCD compared to those who have parents who are skilled and unskilled workers. Also comparing social demographic factors and summarized attitude score of respondents, religion is significantly different from others, as the Christians have a positive attitude towards SCD compared to the Muslims. Also, parents’ highest level of education also plays an important role in the respondents´ attitude towards SCD as respondents whose parents had tertiary education have positive attitude towards SCD. The father´s occupation plays an important role in the practices of respondents towards SCD.

In the comparison of the summarized scores together, it was discovered that a good knowledge brings about a favorable attitude as a p value of <0.001 was gotten in the association between knowledge on SCD and attitude towards SCD. Surprisingly a good knowledge is not equivalent to good practices and a favorable attitude doesn´t mean the respondents would have a good practice towards SCD has a p value of 0.006 and 0.007 was gotten from the association between knowledge on SCD and control practices towards SCD and attitude towards SCD and control practices towards SCD respectively.

In respect to the findings of this study, the respondents have a good knowledge but there is need to improve their attitude and practice towards the disease because having a good knowledge is not only important but the application of the knowledge in a way to stop the spread of the disease is of paramount importance too. To achieve a higher level of good practice and also to reduce the burden of SCD in Nigeria, the school which is the major source of information in this study is hereby advised to focus more on enlightening the students on how to apply their knowledge to stop the spread of SCD. SCD is a disease that brings along with a higher amount of psychological as well as physical pain, secondary school students should therefore be exposed to teachings that broadens their knowledge on the medical as well as psychological aspect of the disease.

## Conclusion

Based on the results of the study which shows a high level of general awareness on SCD and comprehensive knowledge as regards the various genotype related to SCD, tests to be done for genotype screening, as well as dangers and benefits of genotype screening among the respondents is low. If the burden of SCD is to be reduced in the nation, it is therefore important to focus on improving the attitude and practices of the secondary school students as these set of people are the ones to become parents in future and they have the tendency of either reducing or increasing the SCD burden, the school which is the major source of information in this study is hereby advised to focus more on enlightening the students on how to apply their knowledge to stop the spread of SCD. The use of life experiences from members of their peer group living with SCD should be encouraged as teaching aids.

### What is known about this topic

- Secondary school students are usually in relationships that may eventually lead to marriage in future, so issue of pre-marital screening may be of concern, as this may be affected by existing knowledge, attitude and control practice towards SCD;
- Therefore, understanding knowledge about sickle cell inheritance, its health and reproductive health implications as well as behaviour towards individual with SCD particularly among secondary school students is important regarding limiting the spread of the diseases.

### What this study adds

- In respect to the findings of this study, the respondents have a good knowledge but they need to improve on their attitude and practice towards the disease because having a good knowledge is not as important as applying the knowledge in a way to stop the spread of the disease;
- There is a big gap in terms of control practices of SCD, however, with continuous education would improve the control practices.
